# Editorial: Virus Microscopy

**DOI:** 10.1093/jmicro/dfad029

**Published:** 2023-05-22

**Authors:** Yohei Yamauchi

**Affiliations:** Molecular Medicine Laboratory, ETH Zürich, Institute of Pharmaceutical Sciences, Vladimir-Prelog-Weg 3, HCI H305, Zürich 8093, Switzerland

Viruses are medically important obligatory parasites that can impact the health and quality of life of infected patients. Historically, prophylaxis against viral diseases like poliomyelitis and measles became possible by the successful development and roll out of vaccines in the 1950s and 1960s [1]. In recent times antivirals against influenza virus, human immunodeficiency virus type 1 (HIV-1) and hepatitis C virus etc. have proven successful. These therapies block the virus at critical steps of the virus cycle such as viral entry, proteolytic processing, replication and virus release. To discover new antivirals, it is essential that we obtain a deep mechanistic understanding of the virus replication machinery, virus–host cell interactions and the host immune response. In this respect, advanced microscopy approaches are key tools in understanding virus infection biology.

In this Microscopy special issue *Virus Microscopy*, **Coomer and Padilla-Parra** discuss the imaging of HIV-1 virus entry and transmission in organoids of the female genital tract and colorectal tract, using techniques such as two-photon confocal microscopy and light sheet microscopy. **Hu and Noda** describe the structural analyses of Ebola and Marburg virus (*Filoviridae)* nucleocapsid structures as dissected by single particle analysis using cryo-electron microscopy. **Menke et al.** elaborate on techniques such as super-resolution microscopy and quantitative image analysis for investigating the cell entry and infection biology of Orthohantaviruses. Finally, **Petkidis et al.** highlight the power of label-free microscopy including quantitative phase imaging and atomic force microscopy to investigate virus entry and infection.
